# The Impact of Childhood Maltreatment on Intravenous Ketamine Outcomes for Adult Patients with Treatment-Resistant Depression

**DOI:** 10.3390/ph12030133

**Published:** 2019-09-11

**Authors:** Brittany O’Brien, Marijn Lijffijt, Allison Wells, Alan C. Swann, Sanjay J. Mathew

**Affiliations:** 1 Research Service Line, Michael E DeBakey VA Medical Center, Houston, TX 77030, USA; marijn.lijffijt@bcm.edu; 2 Menninger Department of Psychiatry and Behavioral Sciences, Baylor College of Medicine, Houston, TX 77030, USA; alan.swann@bcm.edu (A.C.S.); sjmathew@bcm.edu (S.J.M.); 3 Lone Star Infusion, PLLC, Houston, TX 77079, USA; md@lonestarinfusion.com; 4 Mental Health Care Line, Michael E DeBakey VA Medical Center, Houston, TX 77030, USA

**Keywords:** ketamine, depression, childhood trauma, childhood maltreatment, treatment schedule behavioral sensitization

## Abstract

Childhood maltreatment is associated with a poor treatment response to conventional antidepressants and increased risk for treatment-resistant depression (TRD). The N-methyl-D-aspartate receptor (NDMAR) antagonist ketamine has been shown to rapidly improve symptoms of depression in patients with TRD. It is unknown if childhood maltreatment could influence ketamine’s treatment response. We examined the relationship between childhood maltreatment using the Childhood Trauma Questionnaire (CTQ) and treatment response using the Quick Inventory of Depressive Symptoms–Self Report (QIDS-SR) in TRD patients receiving intravenous ketamine at a community outpatient clinic. We evaluated treatment response after a single infusion (n = 115) and a course of repeated infusions (n = 63). Repeated measures general linear models and Bayes factor (BF) showed significant decreases in QIDS-SR after the first and second infusions, which plateaued after the third infusion. Clinically significant childhood sexual abuse, physical abuse, and cumulative clinically significant maltreatment on multiple domains (maltreatment load) were associated with better treatment response to a single and repeated infusions. After repeated infusions, higher load was also associated with a higher remission rate. In contrast to conventional antidepressants, ketamine could be more effective in TRD patients with more childhood trauma burden, perhaps due to ketamine’s proposed ability to block trauma-associated behavioral sensitization.

## 1. Introduction

Approximately 12.2% of US residents 13 years and older have a lifetime history of recurring major depressive episodes associated with major depressive disorder (MDD) or bipolar disorder (BD) [1]. An estimated 35% of depressed patients have treatment resistant depression (TRD), defined as an inadequate treatment response (<50% improvement in depression severity) to at least two different types of antidepressant medications, the majority of which target monoaminergic neurotransmitter systems [2]. Compared to treatment responders, TRD is associated with a lower quality of life and increased mortality [3,4]. It is important to identify factors that may predict response to specific interventions in order to provide timely, effective treatment.

The N-methyl-D-aspartate receptor (NMDAR) antagonist ketamine is a promising treatment option for TRD [5]. Randomized controlled trials in patients with TRD have consistently shown favorable antidepressant responses to single and repeated subanesthetic doses of ketamine compared to saline or active placebo [6,7,8,9]. Ketamine’s antidepressant effect has been related to pre- and post-synaptic NMDAR blockade, enhancing prefrontal [10] and hippocampal [11] glutamate concentrations which activate the α-amino-3-hydroxy-5-methyl-4-isoxazolepropionic acid receptor (AMPAR), enhancing synaptic plasticity via AMPAR-induced elevation of brain derived neurotrophic factor (BDNF) [12] and activation of the mammalian target of rapamycin (mTOR) signaling pathway [13]. The antidepressant response is often rapid, with patients maintaining substantial gains for up to two weeks [14,15,16].

A risk factor for TRD in adulthood is maltreatment in early life [17,18]. Approximately 12.5% of US children and adolescents have been exposed to sexual abuse, physical abuse or neglect, or emotional abuse or neglect [19]. A history of childhood maltreatment has been associated with a diminished treatment response to conventional antidepressant treatments [17]. No information to our knowledge is available regarding the relationship between childhood maltreatment and ketamine treatment response, although there is emerging support for the efficacy of IV ketamine in reducing symptoms of PTSD in adults [20,21].

In this study, we examine the influence of childhood maltreatment on ketamine treatment response after single and repeated infusions in moderate to severely depressed adults receiving treatment at an outpatient ketamine clinic. We hypothesized that a history of childhood maltreatment would predict an unfavorable treatment response to acute (single infusion) and chronic (repeated infusions) ketamine. Our secondary hypothesis was that ineffectiveness would be related to severity of burden from childhood trauma.

## 2. Results

### 2.1. Patient Characteristics

Table A1 summarizes key demographic, treatment and clinical features of (i) patients who received a single infusion followed by a clinic visit 3–4 or 7 days later for post-infusion assessment (TPIA) (n = 115) and (ii) a subsample of the 115 patients who continued to receive at least four infusions on a twice weekly (every 3–4 days) or weekly schedule (every 7 days) with a baseline or post-infusion assessment prior to each infusion. Patients were included if they had moderate to severe levels of depression at pre-treatment baseline (operationalized as scores >10 on the Quick Inventory of Depressive Symptoms–Self Report, QIDS-SR [22,23]) irrespective of psychiatric disorder. All patients except one reported a diagnosis of major depressive or bipolar disorder; one patient reported PTSD. More than half the sample reported at least one comorbid diagnosis of PTSD, anxiety disorder or pain disorder. Concurrent psychiatric medications spanned 12 different drug classes (mean 2.2 per patient). Patients received on average 0.62 (single infusion) or 0.70 (repeated infusion) mg/kg of IV ketamine per infusion.

### 2.2. Single Infusion

#### 2.2.1. Treatment Effect

Repeated measures general linear models (RM-GLM) tested the effects of ketamine treatment on depressive symptoms. Depressive symptoms were measured with the Quick Inventory of Depressive Symptomatology Self Report (QIDS-SR). Time was included as dependent variable (baseline, post-infusion QIDS-SR). A single ketamine infusion significantly decreased QIDS-SR scores (mean ± SD; baseline 18.63 ± 3.70; post-infusion: 13.12 ± 5.13) (F(1,114) = 175.70, *p* < 0.001, effect size [ES] η^2^_p_ = 0.61; BF > 1.27 × 10^+10^). Of the 115 patients, 19% (n = 22) were responders (≥ 50% reduction of QIDS-SR score from baseline) and 7% (n = 8) achieved remission (QIDS-SR <6).

#### 2.2.2. Effect of Childhood Maltreatment

**Figure A1:**
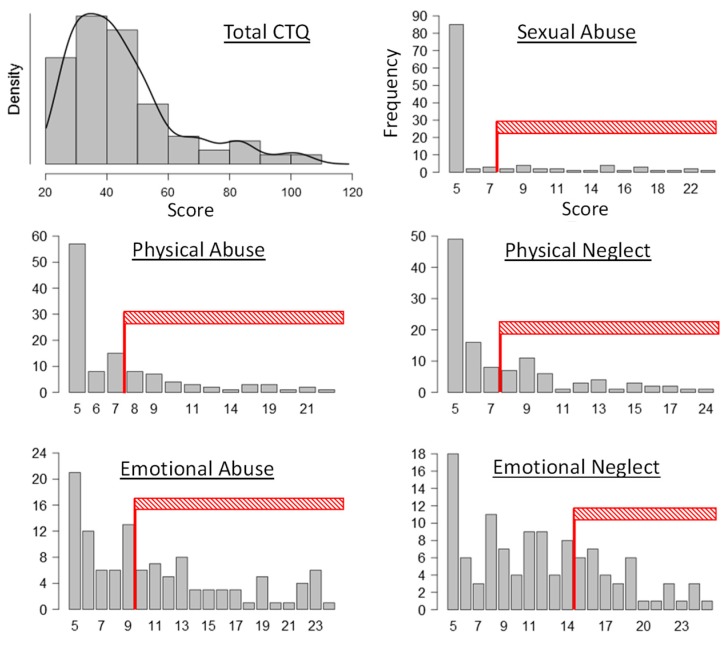
Subject response distributions of total CTQ and CTQ subscales. Clinically significant maltreatment cut-offs for each of the subscales are indicated by the red shaded areas. The number and percentage of subjects are as displayed in Table A1. There is no cut-off available for total CTQ.

Childhood maltreatment was assessed using the Childhood Trauma Questionnaire (CTQ), which provides a total score and subscale scores for five distinct domains of childhood maltreatment: sexual abuse (SA), physical abuse (PA), physical neglect (PN), emotional abuse (EA), and emotional neglect (EN). Table 1 summarizes mean CTQ total and subscale scale scores, the number and percentage of patients with clinically significant maltreatment based on recommended cut-off scores [24], and the number and percentage of patients with significant maltreatment across domains captured by maltreatment loads 0–5 defined as the sum of subscales that met criteria for clinical significance. Figure A1 displays the density and frequency plots for CTQ scores. About two-thirds of the sample had a maltreatment load of 1 or higher.

##### QIDS-SR

We first examined correlations between QIDS-SR change score (baseline minus post-infusion) and CTQ measures. Change score correlated significantly with maltreatment load (r = 0.31, *p* < 0.001; BF_10_ = 29.07), SA (r = 0.29, *p* = 0.001; BF_10_ = 17.22), PN (r = 0.24, *p* = 0.01; BF_10_ = 3.11), total CTQ (r = 0.24, *p* = 0.01; BF_10_ = 2.75) and PA (r = 0.18, *p* = 0.05; BF_10_ = 0.80), but not EA or EN (r < 0.17, *p* > 0.07; BF_10_ < 0.57). Based on r and BF values, we tested effects of maltreatment load, and of SA and PN on treatment response using two separate RM-GLM analyses. p-values are corrected for the two RM-GLM analyses (corrected *p*-value < 0.025 to be significant).

RM-GLM with load as grouping variables showed the effect of time, and a significant time by load interaction (F(1,109) = 3.78, *p* = 0.003, η^2^_p_ = 0.148; BF_10_ = 6.37) (see Appendix H for BF calculation for interactions). Separate ANOVA’s for baseline and follow-up QIDS-SR revealed no significant differences between loads (F[5,109] < 1.71, *p* > 0.13). However, an ANOVA with QIDS-SR change score as dependent variable revealed that patients with load 5 (clinically significant on all 5 subscales) had a larger reduction in QIDS-SR scores from baseline than patients with loads 0, 1 or 3 (t > 3.18, *p* < 0.03; BF_10_ > 5.56); differences were not significant with loads 2 or 4 (t ≈ 2.4, *p* > 0.18; BF_10_ ≈ 2.50).

Subscale RM-GLM analysis included SA and PN as continuous independent variables, showing the effect of time and a time by SA interaction (F(1,112) = 5.37, *p* = 0.022, η^2^_p_ = 0.046). Testing the interaction using SA cut-off scores showed that those with SA ≥8 (n = 24) had a mean decrease in QIDS-SR of 8.08 points (SD = 4.33) compared to 4.82 (4.25) points of those with SA <8 (n = 91) (F(1,113) = 11.07, *p* = 0.001, η^2^_p_ = 0.089; BF_10_ = 25.81). However, this translated to only a 2-point difference at post-infusion (low: 13.62 ± 5.22; high: 11.25 ± 4.37; F(1,113) = 4.15, *p* = 0.04; BF_10_ = 1.41), without a significant difference at baseline (low: 18.44 ± 3.80 high: 19.33 ± 3.28; F(1,113) = 1.11, *p* = 0.38; BF_10_ = 25.81).

##### Response and Remission Rates

Relationships between maltreatment and response and remission rates were examined with X^2^ tests or *t*-tests corrected for 6 comparisons (*p* < 0.0083 for load and five subscales). Neither load nor CTQ subscales were related to response or remission rates after a single infusion.

#### 2.2.3. Influence of Demographic and Treatment Variables on Maltreatment Effects

Table A2 displays demographic, treatment and clinical variables divided by maltreatment load. Loads only differed on CTQ total and subscale scores. A subsequent RM-GLM was performed to determine if effects of maltreatment on treatment response remained significant after controlling for effects of demographic (age, gender) and treatment (ketamine dose, time of post-infusion assessment [TPIA]) variables. Maltreatment load was included as a continuous variable to avoid empty cells when load was included as a grouping factor. Table A3 provides outcomes of that analysis, showing that the interaction remained significant after correcting *p*-values for four RM-GLM analyses.

Outcomes of the tests examining possible moderating effects of self-reported diagnosis and of prescribed psychopharmacological treatment are provided in Table A4 (left-hand column). Demographic, clinical and treatment variables did not affect the time by CTQ maltreatment load interaction.

### 2.3. Repeated Infusions

Table A1 summarizes demographics, treatment variables and diagnostic characteristics of 63 clinic patients who received at least four repeated ketamine infusions on a twice weekly (every 3–4 days) or weekly (every 7 days) treatment schedule. Table 2 summarizes CTQ characteristics. The CONSORT chart in Appendix F displays the reason for the exclusion of 52 patients from the repeated infusion dataset.

#### 2.3.1. Treatment Effect

For a per-protocol analysis using only patients who completed four infusions on a twice- or once-weekly schedule, repeated measures general linear models (RM-GLM) tested the effects of repeated ketamine infusions on depressive symptoms measured with the QIDS-SR. A RM-GLM analysis for QIDS-SR scores across five visits and four infusions showed a significant effect of time (F[4,248] = 97.60, *p* < 0.001, η^2^_p_ = 0.61, BF_10_ = 1.77 × 10^+46^). Table 3 displays outcomes of post-hoc tests correcting p for multiple comparisons, showing significant reductions in QIDS-SR after the first and second infusions. These outcomes were found irrespective of treatment schedule. The Bayes factor (BF) indicates that the evidence for these improvements is strong. Although the improvement in depression after the third infusion was not statistically significant and effect size (d) is low, BF suggests moderate evidence in favor of an improvement. Additional decreases in QIDS-SR scores after the fourth infusion were not significant and BF evidence for improvement was low. Of the 63 patients, 46.03% (n = 29) were responders and 23.81% (n = 15) achieved remission after four infusions.

#### 2.3.2. Effect of Childhood Maltreatment

##### QIDS-SR

We first examined correlations between QIDS-SR change score (QIDS-SR baseline minus visit five) and CTQ variables. Possible effects of childhood maltreatment were examined further with RM-GLM analyses that included CTQ variables that the initial correlation analysis showed to have a significant relationship with QIDS-SR change. QIDS-SR change correlated significantly with maltreatment load (r = 0.427, *p* < 0.001; BF_10_ = 59.89), PN (r = 0.390, *p* = 0.002; BF_10_ = 20.61), total CTQ (r = 0.360, *p* = 0.004; BF_10_ = 9.52), PA (r = 0.359, *p* = 0.004; BF_10_ = 9.25), and SA (r = 0.335, *p* = 0.007; BF_10_ = 5.29). Correlations were not significant for EA (r = 0.137, *p* = 0.284; BF_10_ = 0.275) and EN (r = 0.164, *p* = 0.200; BF_10_ = 0.351). Separate RM-GLM analyses tested for effects of maltreatment load, and of PN, PA and SA. *p*-values are corrected for the two RM-GLM analyses (corrected *p*-value < 0.025 to be significant).

The RM-GLM analysis with load as grouping variable revealed a significant effect of time (F[4,248] = 106.93, *p* < 0.001, η^2^_p_ = 0.65, BF_10_ = 1.77 × 10^+46^) and time by load interaction (F[4,248] = 2.40, *p* = 0.003, η^2^_p_ = 0.17; BF = 6.57) (for extracting BF for interaction terms, see Appendix E and [25]). Load by itself was not significant (F[5,57] = 1.32, *p* = 0.27, η^2^_p_ = 0.10, BF_10_ = 0.213).

**Figure 1:**
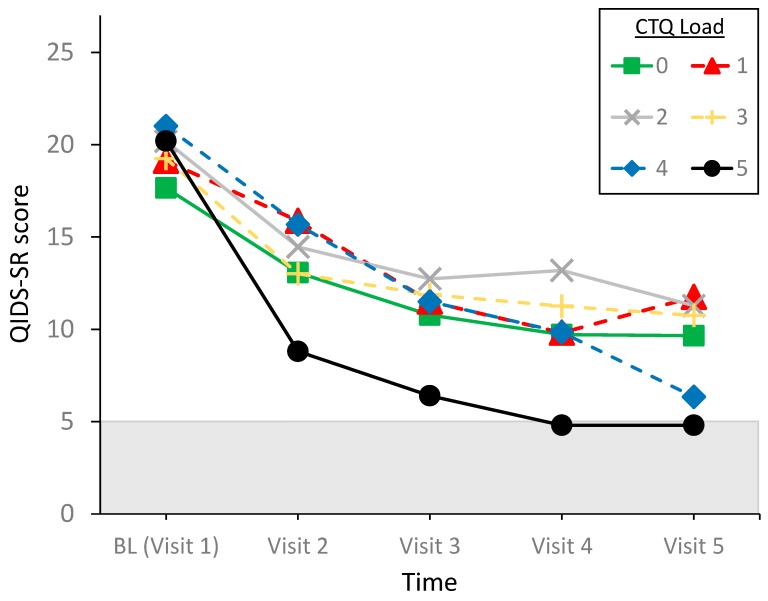
Time by maltreatment load interaction for QIDS-SR. QIDS-SR < 6 indicates remission.

The time by load interaction is displayed in Figure 1. ANOVA’s showed that the load groups did not differ in QIDS-SR score at any of the time points (F[5,57] < 2.08, *p* > 0.81, η^2^_p_ < 0.16; BF_10_ = 0.185 –0.680). By contrast, examination of load effects on QIDS-SR change score revealed Bonferroni-corrected higher change scores for load 4 (QIDS-SR change score = 14.67 ± 4.41) and 5 (15.40 ± 2.30) compared to load 1 (7.38 ± 4.87) (respectively, t = 3.19, *p* = 0.035, d = 1.53; BF_10_ = 2.35, and t = 3.28, *p* = 0.027, d = 1.80; BF_10_ = 3.78), and a trend for a difference between load 0 (8.0 ± 4.85) and load 5 (t = 3.04, *p* = 0.053, d = 1.66; BF_10_ = 2.56). No significant differences were found between the other groups, indicating ketamine could benefit patients with a very high maltreatment load more than patients with a low load.

RM-GLM for CTQ subscales included SA, PA and PN as continuous independent variables. Outcomes showed the significant effect of time, and a significant time by PA interaction (F[4,236] = 5.83, *p* < 0.001, η^2^_p_ = 0.090) with the time by PN interaction approaching significance (F[4,236] = 2.38, *p* = 0.052, η^2^_p_ = 0.039). Main effects of the CTQ subscales (F[1,59] < 2.49, *p* > 0.11) and the time by SA interaction (F[4,236] = 1.57, *p* = 0.18, η^2^_p_ = 0.026) were not significant. As noted by the correlation analysis, those with higher scores on PA have a greater decline in QIDS-SR from baseline to visit five. A final RM-GLM with CTQ total score also revealed the interaction with time (F[4,244] = 3.08, *p* = 0.017, η^2^_p_ = 0.048) with the same effect as that found for PA.

##### Response and Remission Rates

Relationships between maltreatment and response and remission rates at Visit 5 were examined with X^2^ statistics. Outcomes were corrected for multiple comparisons (*p*_cor_ < 0.0083 for CTQ load and the five subscales). Table 4 displays the outcomes of the statistical analyses, revealing significant effects of maltreatment load and PN. BF shows very strong evidence for remission with higher than lower maltreatment load, strong evidence for remission with PN, and moderate evidence for remission with both SA and PA. There were no variable-specific effects on response rate.

**Figure 2:**
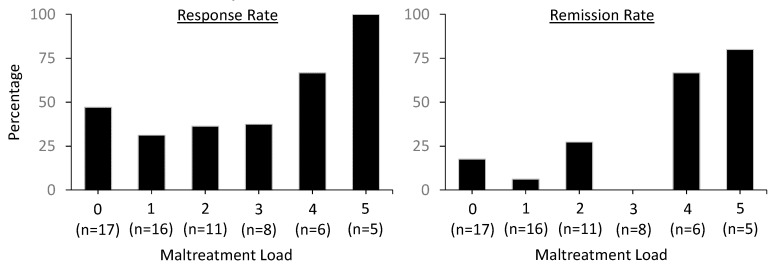
Response and remission rates as a function of CTQ childhood or adolescent maltreatment load.

Exploring the effect of maltreatment load on response and remission rates, Figure 2 displays the percentages of patients who met criteria for response and remission at visit 5. The figure suggests that patients with clinically significant maltreatment on at least four CTQ subscales have a higher rate of response and remission than those with a load of 3 and lower, although the effect of load was significant for remission and not response rate. This indicates that meeting clinical significance of childhood maltreatment on at least four CTQ subscales could predict a higher likelihood of remission after four once- or twice-weekly infusions.

#### 2.3.3. Influence of Demographic and Treatment Variables on Maltreatment Effects

Table A5 displays demographic, treatment and clinical variables divided by maltreatment load. Loads only differed on CTQ total and subscale scores. Examining possible moderating effects of demographic variables (age, gender), treatment variables (ketamine dose, treatment schedule), self-reported diagnosis, and prescribed psychopharmacological treatment on the relationship between childhood maltreatment and ketamine treatment response revealed a minimal influence of those variables on the time by load interaction and the effect of load on remission rate. Those variables did not consistently relate to treatment effect. Effects of diagnosis and of medication on the time by load interaction is provided in Table A4 (right-hand column).

#### 2.3.4. Intent-to-Treat Analysis

We analyzed patients continuing with multiple infusions on a per-protocol basis, excluding patients who did not return to the clinic (n = 32) or who changed to a treatment schedule different that was different from a fixed weekly/bi-weekly (n = 20) (see CONSORT chart in Appendix F). Analyzing our data on an intent-to-treat basis using last-observation-carried-forward, including all 115 subjects, revealed that maltreatment load was not associated with exclusion from the sample (X^2^ = 4.30, *p* = 0.51) and was not related to the reason of exclusion from the sample (X^2^ = 8.73, *p* = 0.56). On the other hand, patients with clinically significant physical neglect were less likely to be excluded (X^2^ = 6.54, *p* = 0.011). No other significant relationships were found.

These outcomes suggest that patients with more severe maltreatment may benefit more from ketamine infusion because they were more likely to follow the fixed twice- and once-weekly treatment schedule than patients with low maltreatment. However, RM-GLM for QIDS-SR with time (five levels), CTQ maltreatment load (five levels) and completer status (two levels, complete vs. exit) revealed the main effect of time on QIDS-SR (F(4,412) = 121.40, *p* < 0.001) and the interaction time x load (F(20,412) = 1.82, *p* = 0.017). An additional interaction between time x completer status appeared (F(4,412) = 3.38, *p* = 0.010). Other main effects or interactions were not significant. Repeating the X^2^ analyses revealed the previously reported effects of load for response rate (X^2^ = 11.60, *p* = 0.041) and for remission rate (X^2^ = 15.19, *p* = 0.010). These outcomes are consistent with those from the per-protocol analyses, suggesting that ketamine could benefit patients with a history of severe compared to low or no maltreatment.

## 3. Discussion

Contrary to our hypotheses, this naturalistic study in TRD patients showed that those with childhood maltreatment not only benefit as much as those without clinically significant maltreatment history, but may benefit more from a single and repeated ketamine infusions. Childhood sexual abuse (single dose) or physical abuse (repeated doses) are also associated with a better treatment response. The effects of maltreatment load on treatment response and on remission rate suggests that the summation of clinically significant childhood maltreatment domains is a better predictor than clinical significance on a specific category of maltreatment.

Outcomes were minimally affected by age, gender, and ketamine dose for single and repeated infusion. For repeated infusion, psychiatric diagnosis (bipolar disorder) and concurrent medication (antipsychotics, hypnotics, atypical antidepressants) could affect outcomes, but the outcomes do not allow speculating how or why these variables influence the effect of maltreatment on ketamine’s treatment response. In general, the outcomes, suggest that ketamine could benefit TRD patients with high maltreatment load across a variety of diagnoses and concurrent treatment, in particular for single infusion. Although women had higher QIDS-SR scores than men irrespective of treatment, we found no evidence of different treatment responses between men and women, extending the lack of gender effects reported in controlled clinical trials using single dose infusion [26] to a clinical setting. Further, the difference in depression between men and women in our study is only 1.61 points on the QIDS-SR, suggesting that this effect is clinically not meaningful.

The relationship between more severe childhood maltreatment and a better treatment response to ketamine could be associated with processes of trauma-induced behavioral sensitization. Thirty years of evidence across species show that trauma (but also uncontrollable stress in general, repeated use of substances of abuse, mood or anxiety episodes, and suicide attempts) could induce sensitization of behavioral, motivational and stress systems, thereby increasing behavioral and physiological reactivity (expression) to subsequent stressors [27,28,29,30]. Induction and expression of behavioral sensitization require activation of N-methyl-D-aspartate receptors (NMDARs) [31,32] albeit via different neural pathways [33,34,35,36,37]. In preclinical models, NMDAR antagonists blocked induction [38] and expression [31] of behavioral sensitization by stress, and in humans with PTSD, a subanesthetic dose of ketamine [20,21] or NMDR antagonist memantine [39] could improve symptoms of hyperarousal and depressive symptoms which are considered expressions of behavioral sensitization. It is, therefore, possible that resistance to conventional antidepressants may be related to expression of sensitization by early stressful events that could be blocked in this population by ketamine. There are currently no validated markers of sensitization, but development of such markers might make it possible to identify and treat “treatment-resistant” depression in a physiologically-based manner.

In addition to effects of childhood maltreatment on treatment response, we also showed that ketamine’s antidepressant effects were similar across infusion schedules (twice or once weekly infusions), with improvements in depression after the first infusion, a further improvement after the second infusion, and perhaps a further improvement after the third infusion before plateauing. A twice weekly infusion schedule for the first three infusions followed by weekly infusions for maintenance may therefore maximize benefit and minimize patient burden.

Several limitations of the current study complicate the interpretation of outcomes. First, the study sample is relatively small, limiting the number of subjects included in the analyses examining the effects of the highest maltreatment loads as well as those examining the influence of medications and comorbid psychiatric diagnoses. Second, demographic and clinical features, such as patient education level, socio-economic status (SES), family history, and history of medication duration and compliance were not available and may have affected outcomes. Third, the CTQ measures childhood maltreatment, but not other sources of trauma such as parental divorce, death of a parent or loved one, or (natural) disasters. It is also a retrospective measure, which may be affected by recall bias, with patients either minimizing or exaggerating actual maltreatment [40]. Fourth, although we accounted for PTSD diagnosis, we did not address possible further moderating effects of adulthood trauma on ketamine treatment response. Finally, outcomes are based on a naturalistic study design which could bias clinical and treatment variables and therefore complicate the generalizability of our findings.

## 4. Materials and Methods

### 4.1. Study Samples

**Figure 3:**
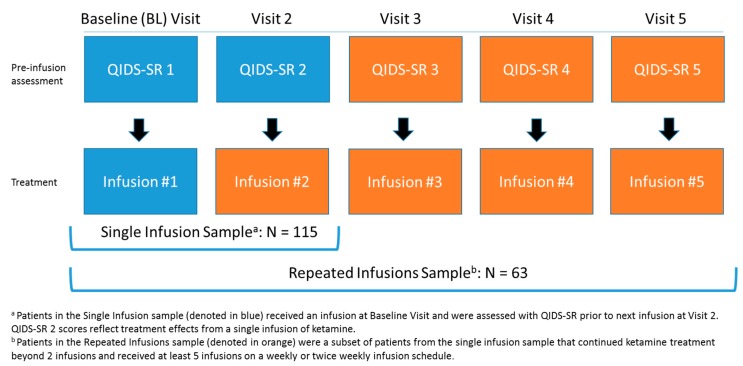
Study samples and schedule of procedures.

This study included adult patients with moderate to very severe depressive symptoms (baseline QIDS-SR > of 10) presenting for treatment at a ketamine treatment clinic. Patients had failed at least one trial of antidepressant medication. The study examined the effects of childhood maltreatment, operationalized as sexual abuse, physical abuse or neglect and emotional abuse and neglect before the age of 18 measured with the Childhood Trauma Questionnaire (CTQ) [41,42], on ketamine’s antidepressant response after a single infusion of ketamine and after at least 4 repeated infusions of ketamine. The first sample of patients (n = 115) received at least 1 infusion of IV ketamine with a post-infusion assessment 3 or 7 days after the infusion. The second sample comprised a subset of patients (n = 63) who continued treatment to receive at least 4 infusions on a twice weekly or weekly basis on Wednesdays and/or Saturdays. The Quick Inventory of Depressive Symptomatology-Self Report (QIDS-SR) [22] was administered at baseline prior to the first infusion, and prior to each subsequent infusion to assess treatment effects. Figure 3 displays the study samples and order of study procedures at each visit.

### 4.2. Administration of IV Ketamine

Treatment infusions took place in a private room equipped with vital sign monitoring and were administered by a board-certified anesthesiologist or anesthetist. Weight based dosing of IV ketamine was delivered over 40 min–2 h as per standard procedures described in numerous publications [6,43]. For nausea, patients were given ondansetron.

### 4.3. Data Set

A waiver of consent was obtained from the Baylor College of Medicine Investigational Review Board (IRB) to analyze de-identified demographic and clinical data from patients who received treatment a ketamine treatment center. Data were collected by clinic staff as part of routine clinical care from April 2016 to April 2019. Researchers received de-identified information in a database.

### 4.4. Materials

The QIDS-SR [22] is a 16-item self-report scale assessing the severity of depressive symptoms. The QIDS-SR assesses all the criterion symptom domains designated by the American Psychiatry Association Diagnostic and Statistical Manual of Mental Disorders-5th edition (APA, 2013) to diagnose a major depressive episode. The QIDS-SR is easy to administer and is sensitive to change. Its psychometric properties have been established in various study samples [22,23].

The CTQ [41,42] is a 28-item self-report scale measuring childhood maltreatment prior to the age of 18. It has been validated in clinical and non-clinical samples, and has sound psychometric properties (internal consistency α > 0.78; test-retest reliability r = 0.88). Twenty-five items assess the presence of abuse or neglect across 5 domains of childhood maltreatment: sexual, physical and emotional abuse, and physical and emotional neglect. Each item is scored on a 5-point Likert scale from never true to very often true, and is in reference to “When you were growing up”. Scores range from 5 to 25 on each of the 5 subscales with higher scores indicating more severe maltreatment. Following previously established guidelines, clinically significant maltreatment in each domain is defined as a score of at least 8 (sexual abuse, physical abuse, physical neglect), 10 (emotional abuse), and 15 (emotional neglect) [24]. We were also interested in the influence of trauma load across maltreatment domains. The CTQ total score does not take into account clinically relevant scores on each subscale. For that reason, we calculated a “maltreatment load” score to denote the total number of domains a patient scored above threshold for clinically significant maltreatment (score 0–5). A higher load indicates more extensive clinically significant childhood maltreatment.

### 4.5. Data Analysis

Ketamine treatment effects on QIDS-SR and the possible influence of childhood maltreatment were tested with repeated measures general linear models (RM-GLM). Time was included as a dependent variable for analyses for a single infusion (baseline, time of post-infusion assessment [TPIA]) and for repeated infusion (baseline [visit 1], visit 2, visit 3, visit 4, visit 5). First, effects of treatment were examined. Second, CTQ variables were included as dichotomous or continuous variables where appropriate. CTQ variables were included only when an initial correlation analysis showed a significant correlation between the CTQ variable and QIDS-SR change score (baseline minus TPIA or visit 5). Finally, demographic characteristics (age, gender), treatment characteristics (ketamine dose, TPIA or treatment schedule), diagnosis and/or concurrent psychoactive medication were included as independent variables to examine possible modulating effects on relationships between maltreatment and treatment response. For all RM-GLM, significant interactions were tested with appropriate follow-up analyses. Relationships between response rate (≥50% reduction from QIDS-SR baseline) and remission rate (QIDS-SR of <6) with demographic, clinical and CTQ variables were tested with X^2^ or *t*-tests where appropriate.

Besides providing p-values to express the rejection of a null hypothesis, extra information is provided by the Bayes factor (BF) about the strength of the evidence in favor of the alternative hypothesis over the null hypothesis (BF_10_) or vice versa [44,45,46].

Data distributions of ketamine absolute dose and dose in mg/kg, SA, PA and PN were normalized with inverse transformations. Statistical outcomes of inversely transformed data are in opposite directions compared to analyses with the original data; we report outcomes in the non-normalized direction (e.g., negative r-values with transformed variables will be presented as positive r-values as if non-normalized). All other variables were normally distributed. All statistical analyses were performed in JASP 0.9.0.1 [45].

## 5. Conclusions

The outcomes from this naturalistic study suggest that in TRD populations with high self-reported childhood maltreatment, ketamine treatment could be considered before other (add-on) antidepressant medications. Outcomes also suggest that the optimal treatment response can be obtained with two or three infusions on a twice-weekly schedule followed by maintenance of the antidepressant response with once weekly ketamine infusions.

## Figures and Tables

**Table 1:** Childhood Trauma Questionnaire (CTQ) maltreatment characteristics of the single infusion study sample (N = 115).

	CTQ Scores		Clinically Significant			Maltreatment Load	
	Mean	SD	N	%		N	%
Total	45.33	18.11	-	-	0	34	29.6
SA ^1^	7.07	4.40	24	20.9	1	26	22.6
PA ^1^	7.57	4.22	34	29.6	2	23	20.0
PN ^1^	7.64	3.68	44	38.3	3	14	12.2
EA	11.10	5.66	56	48.7	4	8	7.0
EN	11.95	5.39	38	33.0	5	10	8.7

CTQ: Childhood Trauma Questionnaire; SA: sexual abuse; PA: physical abuse; PN: physical neglect; EA: emotional abuse; EN: emotional neglect. ^1^ distributions were inversely transformed before statistical analyses.

**Table 2:** CTQ characteristics of repeated infusion sample (N = 63).

	CTQ Scores		Clinically Significant			Maltreatment Load	
	Mean	SD	N	%		N	%
Total	45.92	18.63	-	-	0	17	27.0
SA ^1^	7.27	4.69	15	23.8	1	16	25.4
PA ^1^	7.33	4.51	14	22.2	2	11	17.5
PN ^1^	8.03	3.63	29	46.0	3	8	12.7
EA	11.27	5.74	32	50.8	4	6	9.5
EN	12.02	5.87	21	33.3	5	5	7.9

CTQ: Childhood Trauma Questionnaire; SA: sexual abuse; PA: physical abuse; PN: physical neglect; EA: emotional abuse; EN: emotional neglect. ^1^ distributions were inversely transformed before statistical analyses.

**Table 3:** Post-hoc comparisons corrected for multiple comparisons testing changes in Quick Inventory of Depressive Symptomatology–Self Report (QIDS–SR) scores between clinic visits.

Infusion	Visit	QIDS-SR				t	*p*	d	BF_10_
		Mean	SD	Comparison	Change				
Baseline (BL)	1	19.19	3.71	-	-	-	-	-	-
Infusion 1 (I-1)	2	13.92	4.94	**BL vs. I-1**	**5.27**	**9.12**	**<0.001**	**1.15**	**1.73 × 10+^10^**
Infusion 2 (I-2)	3	11.16	5.32	**I-1 vs. I-2**	**2.76**	**6.50**	**<0.001**	**0.82**	**7.9 × 10^+5^**
Infusion 3 (I-3)	4	10.16	5.81	I-2 vs. I-3	1.00	2.67	0.097	0.34	3.537
Infusion 4 (I-4)	5	9.91	5.52	I-3 vs. I-4	0.25	0.53	0.999	0.07	0.158

Bold: *p* < 0.0125 across 4 comparisons.

**Table 4:** Maltreatment effects on response and remission rates after infusion 4 at visit 5.

	Response Rate				Remission Rate			
	X^2^	df	*p*	BF_10_	X^2^	df	*p*	BF_10_
Load	8.95	5	0.111	1.19	***20.43***	***5***	***0.001***	***41.83***
Any	0.01	1	0.921	0.34	0.49	1	0.485	0.35
SA	3.37	1	0.066	1.18	0.98	1	0.321	0.51
PA	2.41	1	0.120	1.15	*6.81*	*1*	*0.009*	*6.73*
PN	3.43	1	0.064	1.63	***9.14***	***1***	***0.002***	***23.97***
EA	0.41	1	0.521	0.37	1.99	1	0.159	0.68
EN	0.51	1	0.475	0.41	*6.30*	*1*	*0.012*	*5.36*

SA: sexual abuse; PA: physical abuse; PN: physical neglect; EA: emotional abuse; EN: emotional neglect. Bold: *p* < 0.0083 (Bonferroni-corrected). *Italic*: BF > 3 indicating at least moderate evidence of alternative hypothesis over the null hypothesis.

**Figure A2:**
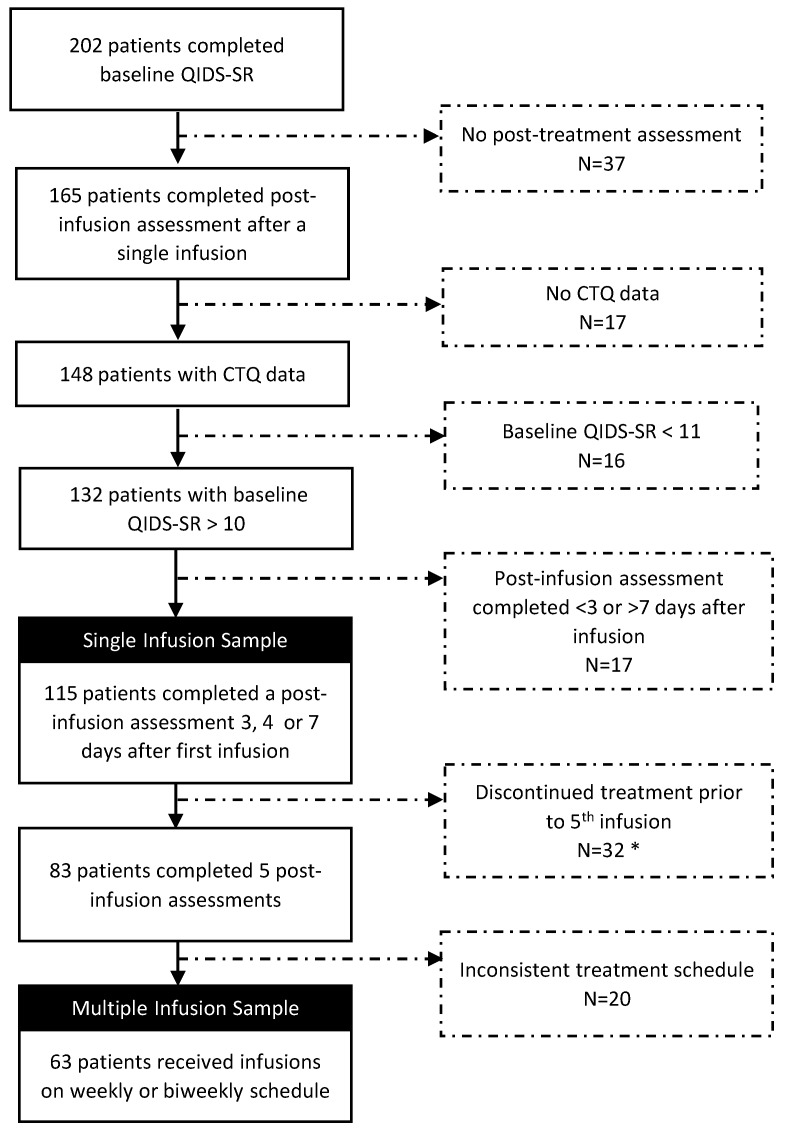
CONSORT chart.

## Appendix A

**Table A1:** Demographic and clinical characteristics.

	Single Infusion (N = 115)			Repeated Infusions (N = 63)		
	Mean	SD	Range	Mean	SD	Range
Age (years)	43.78	14.45	19–76	43.25	13.67	19–70
Weight (kg)	78.18	18.98	47–127	76.87	18.58	47–127
BMI	26.69	5.56	16.3–43.1	26.55	5.41	16.3–43.1
Dose (mg) ^1^	47.07	26.79	15–240	57.59	38.22	20–238
Dose/kg (mg) ^1^	0.62	0.38	0.32–3.02	0.76	0.48	0.37–3.00
QIDS-SR						
Baseline	18.63	3.70	11–26	19.19	3.71	11–26
Total medications	2.2	1.8	0–7	2.3	1.6	0–7
Infusion costs ($)	444.73	50.82	375–565	459.51	56.25	375–565
	**N**	**%**		**N**	**%**	
Time of post-infusion assessment (TPIA)			Infusion Schedule			
Day 3	74	64.3	Twice weekly	41	65.1	
Day 7	41	35.7	Once weekly	22	34.9	
Gender (m/f)	52/63	45.2/54.8		26/37	41.3/58.7	
Diagnosis						
MDD	88	76.5		49	77.8	
BD	26	22.6		14	22.2	
AD	54	47.0		29	46.0	
PTSD	13	11.3		7	11.1	
Pain	7	6.1		5	7.9	
Medication						
Benzodiazepine *	44	38.3		23	36.5	
SSRI	38	33.0		22	34.9	
Anticonvulsant	37	32.2		22	34.9	
SNRI	27	23.5		14	22.2	
Antipsychotic	26	22.6		15	23.8	
AAD	25	21.7		13	20.6	
Psychostimulant	20	17.4		11	17.5	
Hypnotic	12	10.4		8	12.7	
Opioid	9	7.8		8	12.7	
Lithium	9	7.8		4	6.3	
TCA	6	5.2		4	6.3	
Anxiolytic	5	4.4		2	3.2	

AD: anxiety disorder; AAD: atypical antidepressant; BD: bipolar disorder; MDD: major depressive disorder; PTSD: post-traumatic stress disorder; SNRI serotonin-norepinephrine reuptake inhibitor; SSRI: selective serotonin reuptake inhibitor; TCA: tricyclic antidepressant. ^1^ Variables that were normalized with an inverse transformation prior to data analysis. * Benzodiazepine medications were withheld the day of infusion.

## Appendix C

**Table A2:** Demographic, treatment, and clinical characteristics per CTQ maltreatment load for the single infusion.

	CTQ Maltreatment Load						Statistics	
	0	1	2	3	4	5	F/X^2^	*p*
n	34	26	23	14	8	10	-	-
Gender (m/f)	18/16	13/13	11/12	6/8	4/4	0/10	9.48	0.09
Age	41.56 (15.75)	40.77 (14.97)	43.74 (13.20)	51.64 (14.25)	47.63 (13.93)	45.20 (9.22)	1.37	0.24
Weight (kg)	81.41 (17.95)	81.15 (23.85)	74.96 (18.94)	76.21 (15.87)	77.21 (13.59)	70.41 (11.52)	0.84	0.52
Dose (mg) ^1^	51.47 (37.51)	47.02 (15.17)	47.39 (30.18)	45.36 (22.14)	39.38 (7.76)	40 (11.30)	4.33	0.50
Dose (mg/kg) ^1^	0.64 (0.47)	0.59 (0.13)	0.69 (0.58)	0.59 (0.21)	0.51 (0.07)	0.56 (0.09)	3.50	0.62
TPIA (3/7)	24/10	19/7	15/8	9/5	4/4	3/7	7.31	0.20
*CTQ*								
*SA ^1^*	*5.03 (0.17)*	*5.46 (1.17)*	*6.30 (2.75)*	*8.5 (4.80)*	*8 (5.58)*	*17.2 (4.80)*	*51.24*	*<0.001*
*PA ^1^*	*5.38 (0.74)*	*6.42 (2.52)*	*7.22 (2.43)*	*7.57 (3.74)*	*11.63 (5.73)*	*15.6 (6.15)*	*44.04*	*<0.001*
*PN ^1^*	*5.21 (0.48)*	*6.35 (1.67)*	*7.44 (2.95)*	*9.07 (2.73)*	*10.88 (3.31)*	*15.20 (4.32)*	*63.51*	*<0.001*
*EA ^1^*	*6.38 (1.58)*	*9.77 (4.81)*	*11.30 (3.54)*	*16.57 (5.85)*	*16.25 (4.23)*	*18.3 (4.62)*	*64.23*	*<0.001*
*EN*	*7.77 (2.78)*	*9.42 (3.60)*	*13 (3.94)*	*15.79 (4.56)*	*18.5 (3.38)*	*19.7 (3.34)*	*30.80*	*<0.001*
*Total*	*29.77 (3.92)*	*37.42 (5.61)*	*45.26 (3.98)*	*57.50 (9.15)*	*65.25 (13.83)*	*86 (13.65)*	*120.09*	*<0.001*
*Diagnosis (y/n)*								
MDD	25/9	21/5	17/6	11/3	7/1	7/3	1.32	0.93
BD	9/25	5/21	5/18	3/11	1/7	3/7	1.26	0.94
AD	14/20	12/14	11/12	7/7	5/3	5/5	1.34	0.93
PTSD	2/32	3/23	2/21	3/11	0/8	7/3	7.09	0.21
Pain	2/32	1/25	3/20	0/14	0/8	1/9	3.87	0.57
*Medications (y/n)*								
Benzodiazepine	16/18	9/17	7/16	7/7	1/7	4/6	4.93	0.42
SSRI	10/24	10/16	9/14	7/7	1/7	1/9	6.68	0.25
Anticonvulsant	14/20	6/20	11/12	3/11	1/7	2/8	7.67	0.18
SNRI	14/20	5/21	2/21	3/11	1/7	2/8	9.62	0.09
Antipsychotic	8/26	8/18	5/18	2/12	0/8	3/7	4.22	0.52
AAD	7/27	7/19	6/17	2/12	1/7	2/8	1.57	0.91
Psychostimulant	10/24	3/23	4/19	0/14	1/7	2/8	7.17	0.21
Hypnotic	1/33	3/23	3/20	3/11	1/7	1/9	4.09	0.54
Opioid	2/32	2/24	1/22	1/13	2/6	1/9	3.91	0.56
Lithium	2/32	3/23	3/20	0/14	0/8	1/9	3.48	0.63
TCA	3/31	3/23	0/23	0/14	0/8	0/10	6.02	0.30
Anxiolytic	3/31	1/25	1/22	0/14	0/8	0/10	3.11	0.68
Total medications	2.65 (1.69)	2.31 (1.49)	2.26 (2.09)	2 (1.71)	1.13 (1.64)	1.9 (1.97)	1.14	0.34

AD: anxiety disorder; AAD: atypical antidepressant; BD: bipolar disorder; MDD: major depressive disorder; PTSD: post-traumatic stress disorder; SNRI serotonin-norepinephrine reuptake inhibitor; SSRI: selective serotonin reuptake inhibitor; TCA: tricyclic antidepressant; TPIA: time of post-infusion assessment. ^1^ Non-parametric Kruskal-Wallis test because of non-normal distributions. Italics: *p* < 0.05.

## Appendix D

**Table A3:** Repeated measures general linear models (RM-GLM) outcomes of effects of ketamine single infusion on treatment response as a function of CTQ maltreatment load controlling for demographic and treatment variables.

	F(1,108)	*p*	µ^2^_p_
**Main Effects**			
*Time*	*7.74*	*0.006*	*0.067*
*Gender*	*4.10*	*0.045*	*0.037*
*Age*	*4.27*	*0.041*	*0.038*
TPIA	2.94	0.09	0.027
Dose	0.36	0.55	0.003
Load	0.12	0.73	0.001
Interaction effects			
**Time × load**	**7.86**	**0.006**	**0.068**
Time × TPIA	3.03	0.085	0.027
Time × gender	0.12	0.73	0.001
Time × age	0.04	0.85	<0.001
Gender × TPIA	0.16	0.69	0.002
Time × gender × TPIA	0.35	0.56	0.003

TPIA: time of post-infusion assessment. Underlined bold: interaction of interest, *p* < 0.05; italics: *p* < 0.05. RM-GLM analysis that included CTQ maltreatment load, age, gender, time of post-infusion assessment (TPIA; day 3 or day 7) and dose as independent variables showed that QIDS-SR scores decreased with increased age (r = −0.20, *p* = 0.032), and that QIDS-SR across baseline and post-infusion assessment was higher for women (QIDS-SR = 16.60 ± 3.85) than for men (QIDS-SR = 14.99 ± 3.75). The time by load interaction remained significant. X^2^ tests or *t*-tests revealed that response and remission rates were not related to age, dose, gender or TPIA.

## Appendix E

### Influence of Diagnosis and Psychopharmacological Treatment on Maltreatment Effects

Possible effects of diagnosis or medication on time by maltreatment interactions were tested with RM-GLM with baseline and follow-up QIDS-SR as dependent variables, and load as continuous independent variable instead of grouping variable to avoid empty or low populated cells. Each diagnosis or medication was tested separately. Diagnoses and medications did not affect the time by load interaction for single infusion, although for repeated infusion the interaction was no longer significant with the inclusion of antipsychotics, hypnotics or atypical antidepressant medication. No main effects of interactions were revealed for any of the diagnoses or medications.

**Table A4:** Time by maltreatment load interaction for single and for repeated ketamine infusion when correcting for diagnosis or concurrent pharmacological treatment.

		Single Infusion			Repeated Infusions	
	N	F	*p*	N	F	*p*
*Diagnosis*						
MDD	88	11.74	<0.001	49	4.77	0.001
BD	26	11.73	<0.001	14	2.00	0.095
AD	54	12.03	<0.001	29	4.60	<0.001
PTSD	13	12.96	<0.001	7	4.64	0.001
Pain	7	11.83	<0.001	5	4.63	0.001
*Medication*						
SSRI	38	12.15	<0.001	22	3.75	0.006
SNRI	27	10.30	0.002	14	3.69	0.006
Antipsychotic	26	12.84	<0.001	15	1.51	0.199
anticonvulsant	37	12.42	<0.001	22	3.57	0.008
Psychostimulant	20	12.69	<0.001	11	2.94	0.021
Benzodiazepine	44	12.20	<0.001	23	3.96	0.004
Hypnotic	12	10.96	0.001	8	2.32	0.057
AAD	25	11.84	<0.001	13	1.89	0.114
Number of concurrent medications		3.62	0.005		1.68	0.039

AD: anxiety disorder; AAD: atypical antidepressant; BD: bipolar disorder; MDD: major depressive disorder; PTSD: post-traumatic stress disorder; SNRI serotonin-norepinephrine reuptake inhibitor; SSRI: selective serotonin reuptake inhibitor; TCA: tricyclic antidepressant.

## Appendix G

**Table A5:** Demographic, treatment, and clinical characteristics per CTQ maltreatment load for repeated infusion.

	CTQ Maltreatment Load						Statistics	
	0	1	2	3	4	5	F/X^2^	*p*
n	17	16	11	8	6	5	-	-
Gender (m/f)	8/9	8/8	4/7	3/5	3/3	0/5	4.60	0.47
Age	39.94 (16.75)	37.63 (11.26)	44.46 (10.45)	53.13 (11.09)	49.50 (11.19)	46.60 (14.52)	2.07	0.08
Weight (kg)	80.02 (16.95)	82.61 (22.68)	69.85 (20.49)	71.78 (13.24)	72.12 (15.72)	69.67 (10.56)	1.07	0.39
Dose (mg) ^1^	62.83 (43.63)	53.27 (23.61)	54.15 (52.30)	40.61 (14.90)	46.41 (16.34)	45.82 (19.89)	5.14	0.40
Dose (mg/kg) ^1^	0.79 (0.53)	0.65 (0.22)	0.78 (0.69)	0.58 (0.27)	0.66 (0.26)	0.64 (0.20)	2.31	0.81
Schedule (3/7)	11/6	10/6	9/2	5/3	4/2	2/3	2.82	0.73
*CTQ*								
*SA ^1^*	*5.06 (0.24)*	*5.63 (1.41)*	*6.27 (2.61)*	*8.88 (4.94)*	*8.17 (6.40)*	*18.6 (4.62)*	*25.86*	*<0.001*
*PA ^1^*	*5.24 (0.56)*	*6.50 (3.03)*	*5.91 (1.81)*	*6.38 (2.07)*	*10.5 (5.47)*	*18 (6.33)*	*25.58*	*<0.001*
*PN ^1^*	*5.24 (0.56)*	*6.32 (1.62)*	*8.82 (3.57)*	*9.5 (2)*	*11.67 (3.39)*	*14.6 (3.85)*	*40.28*	*<0.001*
*EA ^1^*	*6 (1.5)*	*9.88 (4.76)*	*11.91 (3.75)*	*16.13 (6.33)*	*16.33 (4.63)*	*18.4 (3.91)*	*35.13*	*<0.001*
*EN*	*6.82 (2.22)*	*9.81 (4.02)*	*13.82 (5.23)*	*15.75 (5.85)*	*18.83 (3.82)*	*18.6 (3.51)*	*14.06*	*<0.001*
*Total*	*28.35 (3.26)*	*38.13 (5.81)*	*46.73 (4.45)*	*56.63 (10.91)*	*65. 5 (15.45)*	*88.2 (7.53)*	*66.99*	*<0.001*
*Diagnosis (y/n)*								
MDD	13/4	12/4	9/2	6/2	5/1	4/1	0.35	0.99
BD	43/13	4/12	2/9	2/6	1/5	¼	0.35	0.99
AD	7/10	7/9	6/5	2/6	5/1	2/3	5.37	0.37
PTSD	2/15	3/13	1/10	1/7	0/6	0/5	2.39	0.79
Pain	1/16	1/15	2/9	0/8	0/6	1/4	3.94	0.56
*Medications (y/n)*								
Benzodiazepine	7/10	6/10	3/8	4/4	1/5	2/3	2.25	0.81
SSRI	5/12	7/9	5/6	4/4	1/5	0/5	5.68	0.34
Anticonvulsant	7/10	5/11	6/5	2/6	1/5	1/4	3.97	0.55
SNRI	7/10	3/13	0/11	2/6	1/5	1/4	6.95	0.23
Antipsychotic	4/13	7/9	2/9	1/7	0/6	1/4	6.18	0.29
AAD	3/14	4/12	3/8	2/6	1/5	0/5	2.03	0.85
Psychostimulant	6/11	1/15	2/9	0/8	1/5	1/4	6.87	0.23
Hypnotic	0/17	2/14	2/9	3/5	1/5	0/5	8.02	0.16
Opioid	2/15	2/14	1/10	1/7	2/4	0/5	3.18	0.67
Lithium	1/16	1/15	1/10	0/8	0/6	1/4	2.66	0.75
TCA	1/16	3/13	0/11	0/8	0/6	0/5	6.18	0.29
Anxiolytic	1/16	1/15	0/11	0/8	0/6	0/5	1.88	0.87
Total medications	2.59 (1.54)	2.63 (1.46)	2.27 (1.95)	2.38 (1.51)	1.50 (1.76)	1.40 (2.07)	0.80	0.55

AD: anxiety disorder; AAD: atypical antidepressant; BD: bipolar disorder; MDD: major depressive disorder; PTSD: post-traumatic stress disorder; SNRI serotonin-norepinephrine reuptake inhibitor; SSRI: selective serotonin reuptake inhibitor; TCA: tricyclic antidepressant; TPIA: time of post-infusion assessment. ^1^ Non-parametric Kruskal-Wallis test because of non-normal distributions. Italics: *p* < 0.05.

## Appendix H

To calculate BF for interaction terms, we provide an example from the text of the interaction between time and maltreatment load. BF is extracted from the output in JASP [28]. Comparing the interaction model (combination of time main effects model + load main effects model + time x load interaction, BF = 4.215 × 10^+46^) with the main effects model (time + load main effects, BF = 6.419 × 10^+45^) showed that the interaction model was preferred over the main effects model by BF = 6.57 (1/[6.419 × 10^+45^/4.215 × 10^+46^]).
